# Poly(Lactic Acid) Composites

**DOI:** 10.3390/ma12213586

**Published:** 2019-10-31

**Authors:** Mosab Kaseem

**Affiliations:** Department of Nanotechnology and Advanced Materials Engineering, Sejong University, Seoul 05006, Korea; mosabkaseem@sejong.ac.kr

**Keywords:** polylactic acid composites, fabrication, dispersion, morphology, mechanical properties, thermal properties, applications

## Abstract

Polylactic acid-based materials have gained great interest within the scientific community due to their biodegradability, good performance, and suitability for a number of applications. Therefore, this Special Issue “Poly(lactic acid) Composites” is proposed to cover the important advances in poly (lactic acid) composites, ranging from their design, fabrication, and material properties to the potential applications of these materials. Therefore, we believe that the present Issue can convey beneficial information to scientists and engineers in numerous fields, including polymer science and biomedical engineering.

## Introduction

Poly(lactic acid) (PLA) is a biodegradable polymer that has been widely used in various industrial fields owing to its biocompatibility, high Young’s modulus, and high tensile strength [1,2]. PLA can be processed by conventional processing methods, such as injection molding and extrusion. In comparison to petroleum-based polymers, such as polyethylene, polypropylene, polystyrene, and polyethylene terephthalate [3], the mechanical properties of PLA are appealing, particularly its Young’s modulus, making it an excellent substitute for petroleum-based polymers in short-time packaging. Despite the abovementioned positive features, PLA is a brittle polymer with poor toughness, representing one of its main limitations for the sustainable development of PLA [4]. In addition, the usage of PLA, especially in the biomedical field, is still limited by its low biodegradability and hydrophobicity [5].

To overcome the drawbacks of PLA described above and develop advanced materials for a variety of applications, several approaches, such as copolymerization, polymer blending, and polymer compositing, have been proposed by several research groups [6,7,8]. Among them, the polymer compositing method, which includes the reinforcement of PLA with fillers or nanofillers to form so-called PLA composite materials, has attracted tremendous interest as an easy and cheap method for promising materials for a wide variety of applications [5]. Accordingly, several organic and inorganic additives, such as silica, alumina, zinc oxide, calcium carbonate, magnetite, starch, wood, carbon nanotube (CNT), and nanocellulose, have been used to enhance the performance of the PLA matrix [9]. Such additives can be divided into natural, semi-synthetic, and synthetic based on their origin [5]. For example, due to their remarkable features, such as high mechanical, thermal, and electrical properties, PLA/CNT composites are considered to be promising materials for medical and industrial applications [10]. In addition, PLA composites with biodegradable fillers, such as starch and nanocellulose, may lead to enhanced properties while still meeting environmental requirements. For example, PLA/nanocellulose composites are considered “green composites”, which are of significance with regard to environmental issues since both PLA and nanocellulose are biodegradable and renewable [11]. Moreover, the presence of nanocellulose can lead to improvements in the mechanical, thermal, and thermomechanical properties of the PLA matrix [11,12]. PLA composites with biodegradable fillers, therefore, are expected to maintain a sustainable, productive society that produces waste materials at a rate at which they can be reabsorbed by the environment. However, the self-aggregation and dispersibility of the fillers should be considered during the fabrication of PLA composites in order to obtain high performance composites that are suitable for a variety of purposes in the fields of packaging energy, biomedicine, optoelectronics, etc. Based on the on-going research on polymer composites, we believe that much attention paid to PLA composites has been broadened not only by the polymer communities but also by the overall materials of societies across research borders. Therefore, we hope that the contributions to the present Issue can effectively support the research on the PLA composites.
